# The non-visual opsins expressed in deep brain neurons projecting to the retina in lampreys

**DOI:** 10.1038/s41598-020-66679-2

**Published:** 2020-06-15

**Authors:** Emi Kawano-Yamashita, Mitsumasa Koyanagi, Seiji Wada, Tomoka Saito, Tomohiro Sugihara, Satoshi Tamotsu, Akihisa Terakita

**Affiliations:** 1 Department of Biology and Geosciences, Graduate School of Science, Osaka City University, 3-3-138 Sugimoto, Sumiyoshi-ku, Osaka, 558-8585 Japan; 2 Department of Chemistry, Biology, and Environmental Science, Faculty of Science, Nara Women’s University, Kitauoyanishi-machi, Nara, 630-8506 Japan

**Keywords:** Visual system, G protein-coupled receptors, Neurophysiology

## Abstract

In lower vertebrates, brain photoreceptor cells express vertebrate-specific non-visual opsins. We previously revealed that a pineal-related organ-specific opsin, parapinopsin, is UV-sensitive and allows pineal wavelength discrimination in lampreys and teleost. The Australian pouched lamprey was recently reported as having two parapinopsin-related genes. We demonstrate that a parapinopsin-like opsin from the Japanese river lamprey exhibits different molecular properties and distribution than parapinopsin. This opsin activates Gi-type G protein in a mammalian cell culture assay in a light-dependent manner. Heterologous action spectroscopy revealed that the opsin forms a violet to blue-sensitive pigment. Interestingly, the opsin is co-localised with green-sensitive P-opsin in the cells of the M5 nucleus of Schober (M5NS) in the mesencephalon of the river and brook lamprey. Some opsins-containing cells of the river lamprey have cilia and others an axon projecting to the retina. The opsins of the brook lamprey are co-localised in the cilia of centrifugal neurons projecting to the retina, suggesting that cells expressing the parapinopsin-like opsin and P-opsin are sensitive to violet to green light. Moreover, we found neural connections between M5NS cells expressing the opsins and the retina. These findings suggest that the retinal activity might be modulated by brain photoreception.

## Introduction

In most vertebrates, light is captured through light-sensitive proteins called opsins, and light information is utilized not only for vision but also for non-visual functions, such as light entrainment of circadian rhythms^1^. Thousands of opsins have been identified in a wide variety of metazoans and are classified into several groups^2^. In vertebrates, the members of Opn3, Opn4 and Opn5 groups are distributed to ocular and extraocular cells and are involved in non-visual functions. Further, four types of vertebrate-specific non-visual opsins, which are close to but clearly distinguished from vertebrate visual opsins, are found in non-mammalian vertebrates, including lampreys, teleost, frogs, lizards and birds^2^. These opsins are categorised into vertebrate non-visual opsin group: pinopsin^3,4^, parapinopsin^5^ and parietopsin^6^ are characterised as opsins of pineal and related organs, and vertebrate ancient opsin (VA/VAL opsin)^7,8^ are distributed to the brain tissues, including the diencephalon and mesencephalon, in addition to the retina^7–10^. Based on the distribution, these vertebrate non-visual opsins are thought to be involved in the photoreceptive functions of the pineal and related organs and of the deep brain photoreceptors in non-mammalian vertebrates^11^.

Previously, we investigated parapinopsin as a vertebrate non-visual opsin model to obtain a clue to relevance of the molecular properties of a non-visual opsin to its physiological functions^11–16^. We found that parapinopsin, which is first found in catfish pineal and parapineal organs^5^, is a UV-sensitive opsin and is involved in the pineal wavelength discrimination of the river lamprey^14^. Although phylogenetically close, the molecular properties of parapinopsin differs from those of vertebrate rod and cone visual opsins^14^. Parapinopsin converts to a stable photoproduct with an absorption maximum in the visible region and reverts to its original dark state by subsequent light absorption, showing interconvertibility or a bistable nature, similar to invertebrate (protostome) visual opsins, melanopsins and Opn5s^17–23^. Recently, we found that the bistable nature of parapinopsin, providing a UV-sensitive dark state and a visible (green) light-sensitive photoproduct, achieves colour opponency involving parapinopsin (PP1) alone in pineal photoreceptor cells of zebrafish^24^. In teleost, parapinopsin has diversified into two types: UV-sensitive PP1 and blue to green-sensitive PP2, involved in the two major functions of the teleost pineal organs, the wavelength discrimination and light regulation of melatonin secretion, respectively^25^. Parapinopsins are the key opsins for non-visual functions.

Recently, two opsin-encoding genes similar to parapinopsins were identified from the genome of the Australian pouched lamprey (*Geotria australis*)^26^. Interestingly, the phylogenetic tree in a previous report^26^ indicates a possibility that pouched lamprey parapinopsins and other parapinopsins composed of river lamprey parapinopsin and gnathostomes parapinopsins are paralogous. Thus, the river lamprey genome might also encode an orthologue of pouched lamprey parapinopsins, in addition to river lamprey parapinopsin (“classical parapinopsin”) that we previously characterised. Therefore, investigation of the functional differences among multiple parapinopsin-related opsins in the river lamprey is of interest.

In this study, we isolated a cDNA of a parapinopsin-like opsin from the Japanese river lamprey (*Lethenteron camtschaticum*) and analysed its molecular properties. We also investigated the expression patterns for the parapinopsin-like opsin in the Japanese river lamprey and a closely related species, the brook lamprey (*Lethenteron reissneri*). We revealed that the parapinopsin-like opsin is violet to blue-sensitive, unlike the known UV-sensitive parapinopsins from lamprey, teleost and green iguana^14,25,27^. Further, the parapinopsin-like opsin is co-localised with P-opsin, which is a homologue of VA/VAL opsin^28^, in the M5 nucleus of Schober in the lamprey mesencephalon, which contains centrifugal neurons projecting to the retina.

## Results

**Figure 1:**
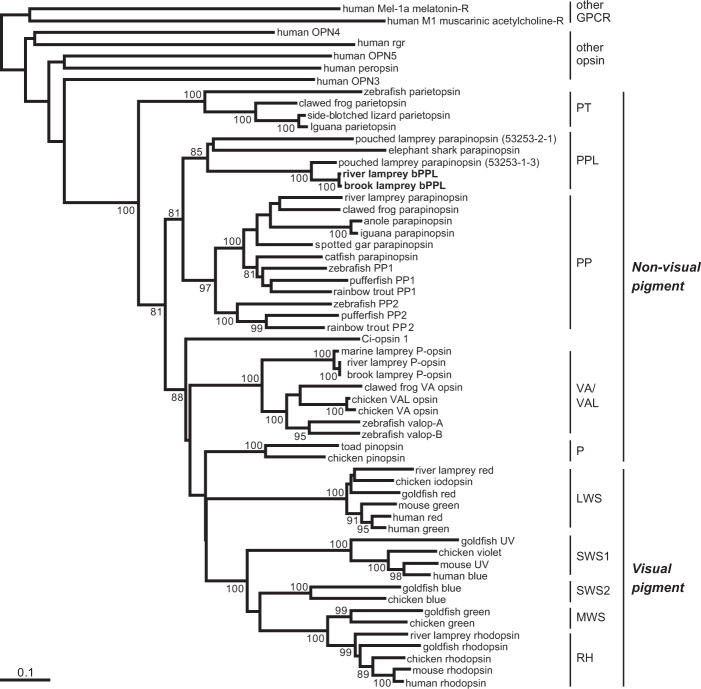
Phylogenetic position of lamprey bPPL. River and brook lamprey bPPLs are classified into PPL, but not PP groups. P: pinopsin, PP: parapinopsin, PPL: parapinopsin-like, PT: parietopsin, VA/VAL: VA/VAL opsin. Bootstrap probabilities (≥80%) are indicated at each branch node. The scale bar indicates 0.1 substitutions per site.

**Figure 2:**
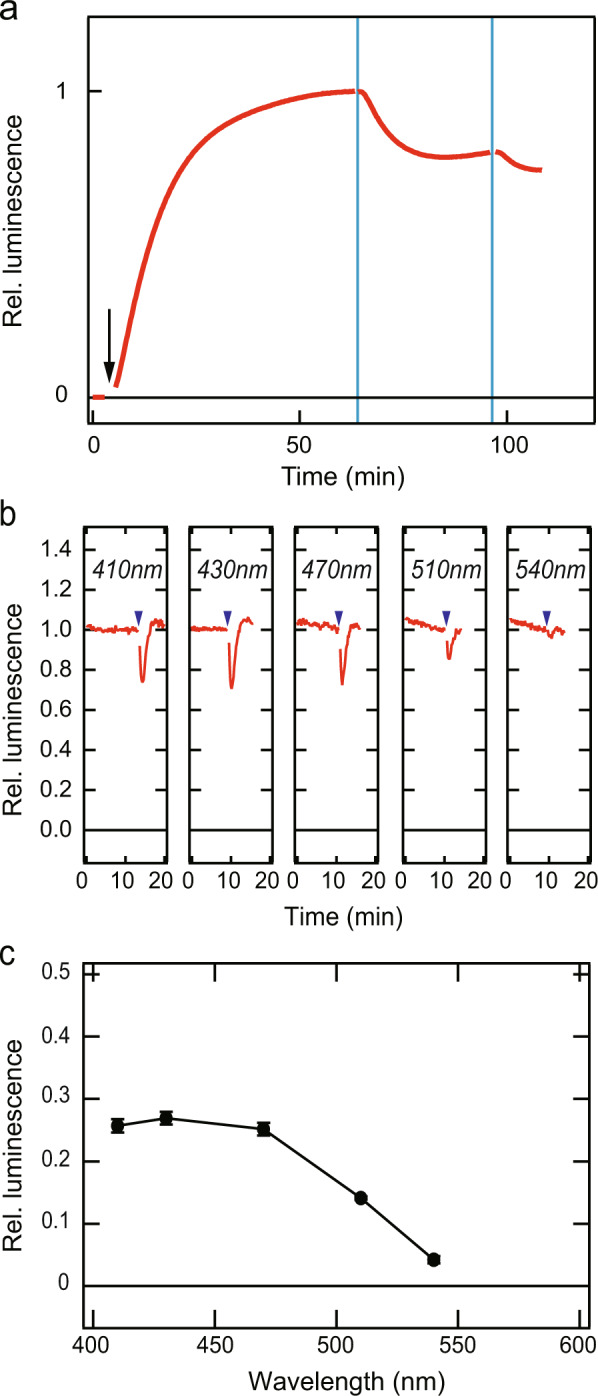
Light-induced cAMP concentration changes in river lamprey bPPL-expressing HEK293S cells and its relative response curve. (**a**) Blue light-dependent decrease in luminescence signal representing the cAMP level as observed in the lamprey bPPL-expressing HEK293S cells. The arrows and vertical lines indicate forskolin treatment and blue-light irradiation (450 nm), respectively. (**b**) The change in the relative luminescence of cells expressing lamprey bPPL upon light irradiation with matched light stimuli of five distinct wavelengths (410, 430, 470, 510 and 540 nm). The arrowheads indicate the timing of light irradiation. Note that cAMP changes were measured without adding forskolin to avoid forskolin-dependent baseline changes. (**c**) The relative response curve of lamprey bPPL. Changes of relative luminescence are shown as the relative responses (black circles). Lamprey bPPL is a violet to blue-sensitive pigment, which exhibited the maximal responses between 410 and 470 nm. Data are expressed as mean ± SE, N = 3.

We isolated a cDNA encoding an opsin similar to parapinopsin, which is UV-sensitive and is involved in the pineal wavelength discrimination^14^, from the lamprey brain by PCR amplification. The molecular phylogenetic tree of vertebrate visual and non-visual opsins, including parapinopsins, indicated that the isolated opsin forms a cluster with one of two pouched lamprey parapinopsins (parapinopsin 53253-1-3^26^, accession no. ANV21069), but not with the well-characterised UV-sensitive ‘classical’ parapinopsins^14^ (Fig. 1). The isolated opsin is referred to as bPPL to reflect its spectral property, violet to blue sensitivity as described below (see Fig. 2). The members of the two clusters are in close paralogous relationship and are referred to as the PP group for the classical parapinopsins and PPL (PP-like) group including bPPL for the parapinopsin-like opsins.

To investigate photosensitivity, we expressed the isolated parapinopsin-like opsin, bPPL, in HEK293S cells. We tested whether bPPL light-dependently decreases cAMP level *via* the activation of Gi-type G protein using GloSensor, a cAMP-sensitive luciferase, similar to the river lamprey parapinopsin (PP group) in cultured cells^13^. The cAMP levels, which were initially increased by forskolin, were decreased by blue-light irradiation in the cultured cells expressing bPPL (Fig. 2a), demonstrating that bPPL activated G protein, possibly Gi-type G protein, which mediated this decrease. We then conducted heterologous action spectroscopy using GloSensor assays to investigate the relative responses of bPPL to light of different wavelengths, in accordance with our previously described method^29^, because a sufficient amount of recombinant bPPL to measure an absorption spectrum was not obtained. Maximal responses were observed with 410–470 nm light, showing that bPPL is most sensitive to violet to blue-light (Fig. 2b,c). Thus, the absorption property of bPPL is different from that of PP group parapinopsins, characterised as UV-sensitive opsins^14^.

We determined a rough distribution for bPPL in the river lamprey brain, including pineal organs (Supplementary Fig. S1). We analysed the mRNA levels of bPPL and parapinopsin in five brain regions: the pineal organ, telencephalon, diencephalon, mesencephalon, and rhombencephalon. Interestingly, bPPL gene expression was found in the diencephalon and mesencephalon in addition to the pineal organ, although the levels of expression were much lower than parapinopsin in the pineal organ. As parapinopsin is abundantly distributed to the pineal organs-containing region, bPPL might be involved in an extraocular photoreception different from that of parapinopsin.

**Figure 3:**
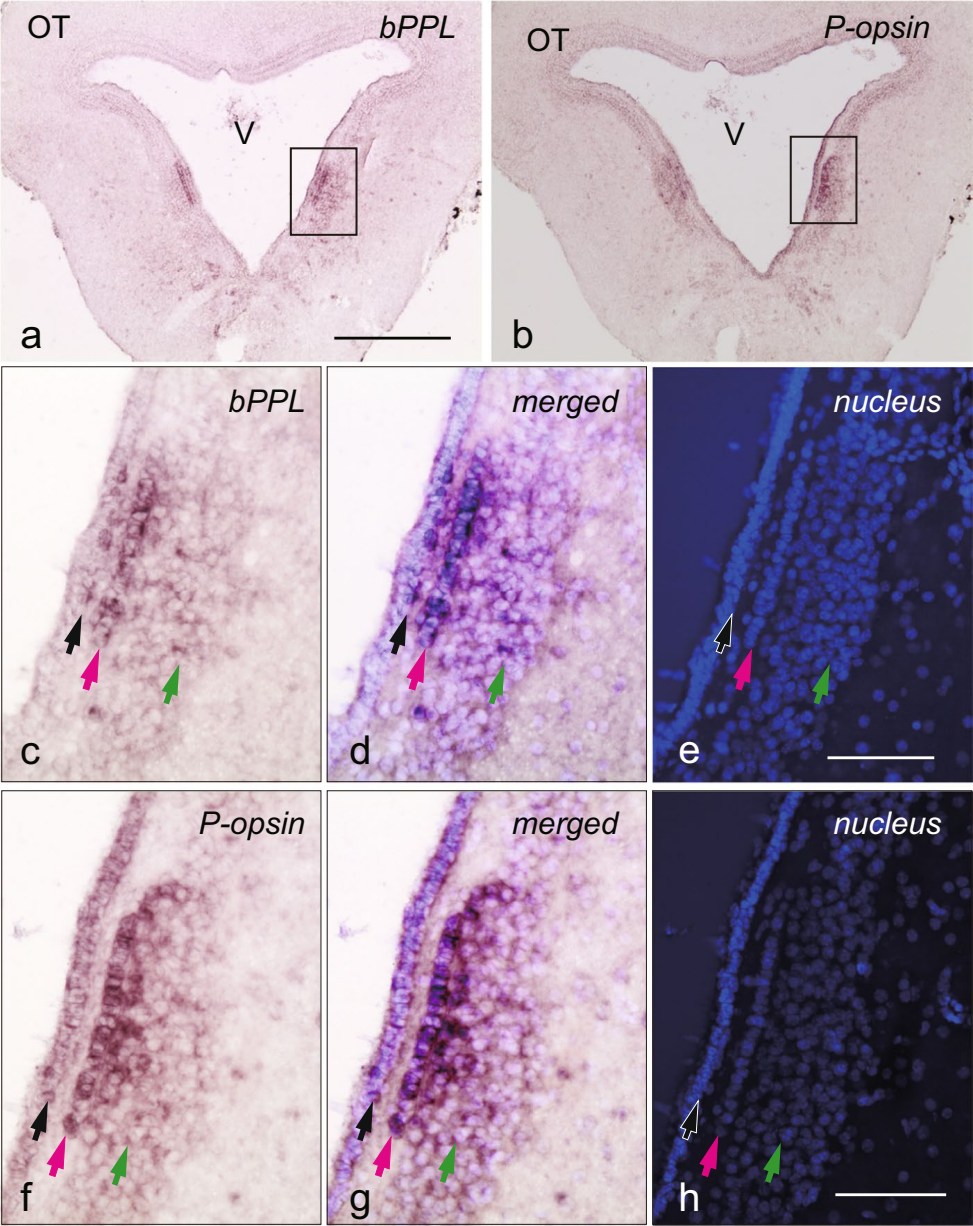
*In situ* hybridisation of bPPL and P-opsin in the river lamprey mesencephalon. *In situ* hybridisation with bPPL (**a**) and P-opsin (**b**) antisense probes demonstrates that both bPPL and P-opsin are expressed in the M5 nucleus of Schober (M5NS). Images of bPPL and P-opsin (boxes in panel a, b) with higher-magnification are presented in panel c–e and f–h, respectively. Panels c and f present *in situ* hybridisation images of bPPL and P-opsin, respectively. Nuclear staining images are presented in panels e and h. Merged images are presented in panels d and g. Cells in Area I are distributed within the periventricular region along the third ventricle (black arrows). Cells in Area II form a line along the third ventricle (magenta arrows). Cells in Area III are distributed in the most lateral region and over a wide area (green arrows). OT: optic tectum, V: third ventricle. Scale bars = 500 μm (**a**) and 100 μm (**e**,**h**).

We then conducted *in situ* hybridisation to clarify the localisation of bPPL in the river lamprey brain. We found clear signals in the mesencephalon (Fig. 3a), but not in the diencephalon or pineal organ (Supplementary Fig. S2). bPPL was expressed in the M5 nucleus of Schober (M5NS)^30,31^ of the lamprey mesencephalon, especially in the cells of three distinct areas of M5NS: the inside of the periventricular region along the third ventricle (Area I), the liner narrow area close to the further side of Area I from the third ventricle (Area II) and the most lateral region in clusters of the bPPL-expressing cells (Area III) (Fig. 3c–e). Further, we examined the expression of P-opsin, a lamprey VA/VAL opsin, which is known as a vertebrate non-visual opsin expressed in teleost diencephalon and mesencephalon^9^. P-opsin was also expressed in the cells in Areas I, II and III of M5NS (Fig. 3b, 3f–h), indicating an expression profile similar to that of bPPL.

**Figure 4:**
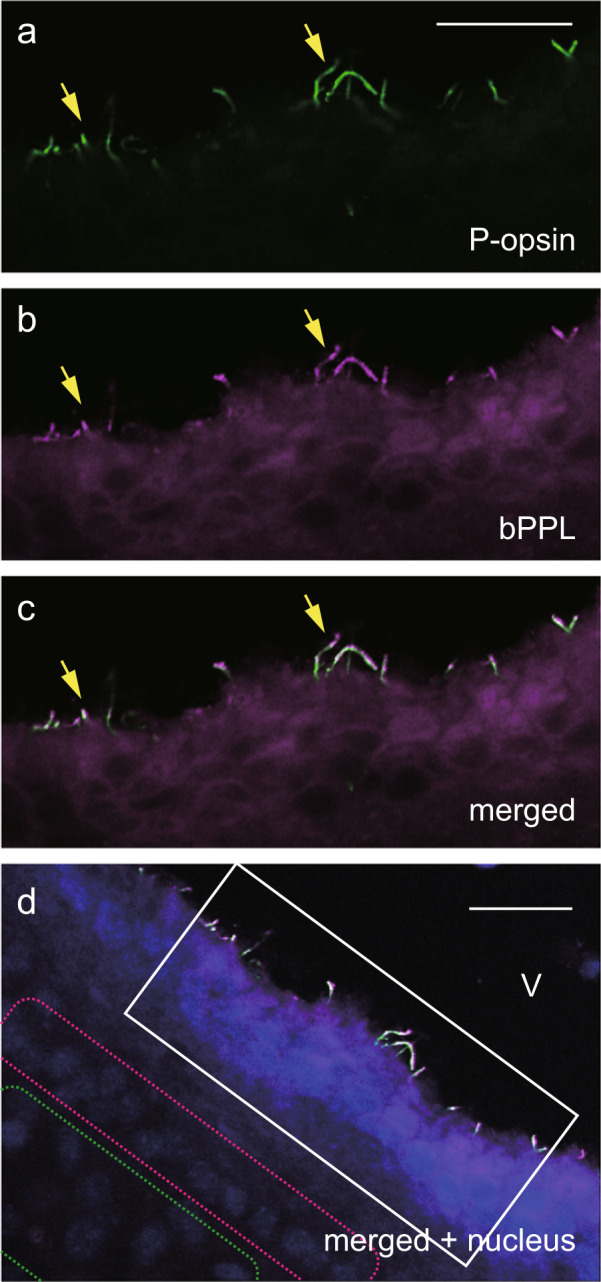
Immunohistochemical co-localisation of bPPL and P-opsin in M5NS of the river lamprey. P-opsin (a, green) and bPPL (b, magenta) are co-localised in the cilia structure of the M5NS (yellow arrows, merged image in panel c) by confocal imaging. The region containing Area I is presented in panels a–c. Nuclear staining is shown in panel d, together with the merged image shown in panel c. The white box in panel d presents the position of panels a–c (Area I). The positions of Areas II and III are presented in magenta and green dotted squares, respectively. V: third ventricle. Scale bars = 25 μm (**a**,**d**).

Next, we generated antisera specific to bPPL and P-opsin (Supplementary Fig. S3) and investigated the co-localisation of bPPL and P-opsin within the M5NS cells. Both the anti-bPPL and anti-P-opsin antisera specifically stained cilia along the third ventricle of M5NS (Fig. 4a,b, Supplementary Fig. S4). Through double immunostaining, confocal images revealed that bPPL is co-localised with P-opsin, specifically in some cilia (Fig. 4c,d). This finding, together with the understanding that retinal and pineal photoreceptor cells developed photoreceptive segments (outer segments) originated from cilia, suggests that cilia expressing both opsins might serve as photoreceptive segments of M5NS cells.

**Figure 5:**
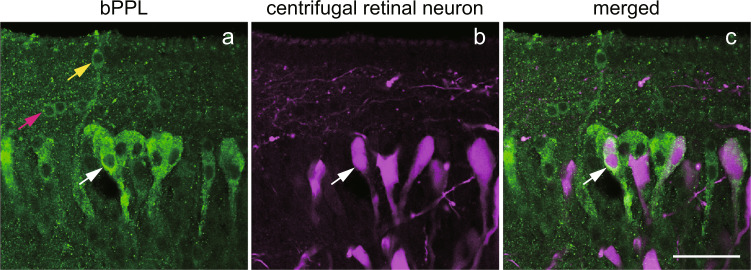
Immunohistochemical localisation of bPPL in centrifugal retinal neurons of M5NS in the river lamprey. The bPPL immunoreactivity (**a**, green) was found in centrifugal retinal neurons (**b**, magenta) in the M5 nucleus of Schober by confocal imaging. The merged image is presented in panel c. Yellow, magenta and white arrows show cells in Areas I, II and III, respectively. Scale bar = 50 μm.

We previously revealed that at high concentration, the antibodies against opsins stained the processes of vertebrate retinal and pineal photoreceptor cells, in addition to outer segments expressing a large number of opsins^32^. Antiserum against bPPL at high concentration stained the cell bodies and basal parts of bPPL-expressing cells weakly but clearly. The immunoreactivity delimited the shape of bPPL-expressing cells, whereas the high concentration of anti-P-opsin antiserum did not. bPPL-expressing cells identified by *in situ* hybridisation (Fig. 3) were also immunohistochemically observed in Areas I, II and III (Fig. 5a). Stained cells in Area I were bipolar in shape and possessed cilia with terminals protruding into the third ventricle, indicating that the cilia contain both bPPL and P-opsin. The cells in Area II exhibited weaker immunoreactivity and formed a line along the third ventricle, and the cells in Area III were larger in size and their basal structure extended laterally.

Previous studies reveal that the so-called centrifugal retinal neurons, directly projecting from the brain to the retina, were present in M5NS^33,34^. We next compared the localisation of bPPL-expressing cells and the centrifugal retinal neurons. It has been found that centrifugal retinal neurons, stained by the retrograde labelling, were localised in M5NS (Fig. 5b), and their shapes were similar to those of cells expressing bPPL in Area III (Fig. 5a). Double staining revealed that bPPL was localised in some centrifugal retinal neurons (Fig. 5c), suggesting that at least some bPPL-expressing cells in Area III may serve as the centrifugal retinal neurons.

**Figure 6:**
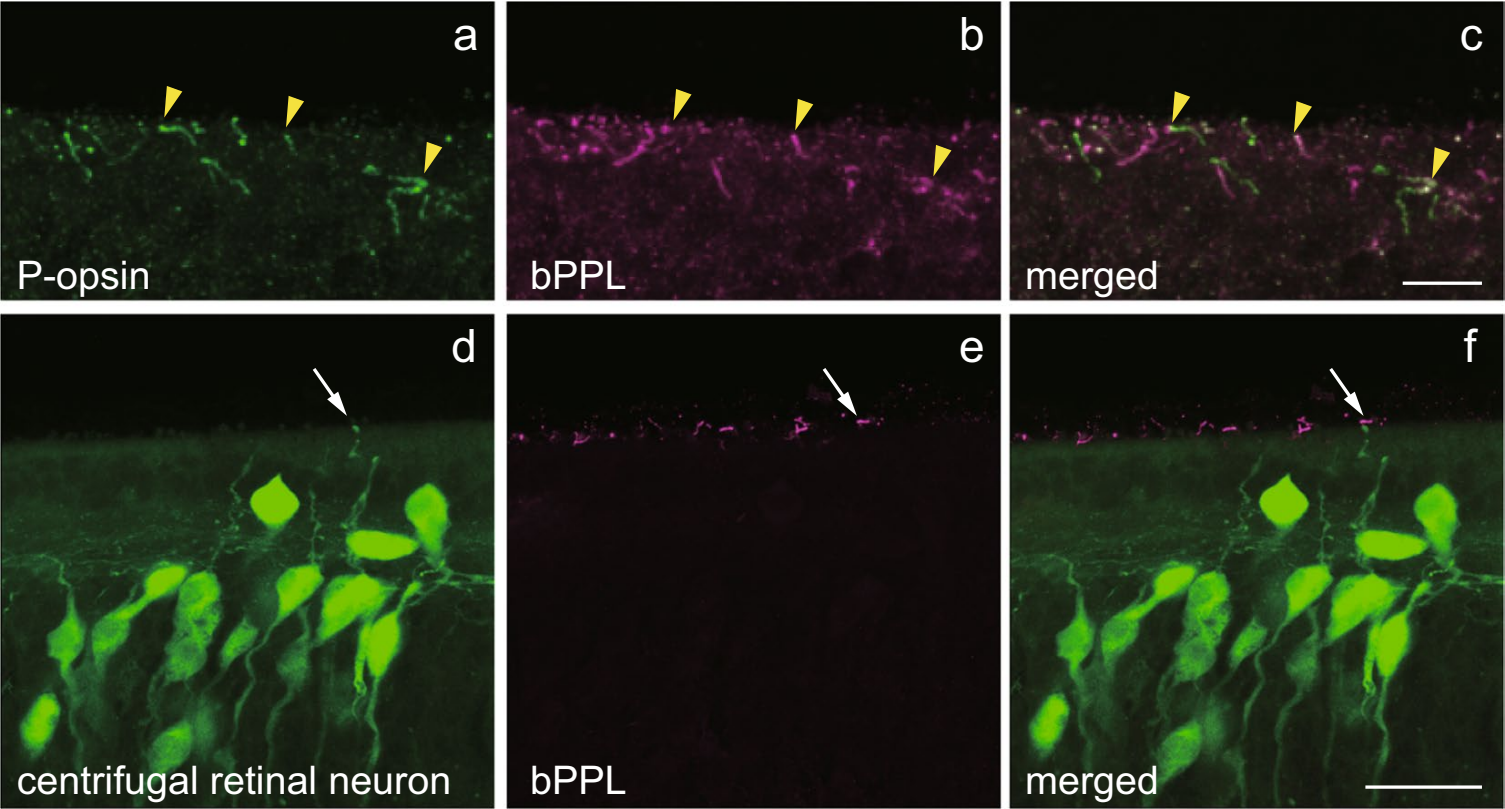
Immunohistochemical localisation of bPPL in the centrifugal retinal neurons of M5NS in brook lamprey. In brook lamprey, P-opsin (**a**, green) and bPPL (**b**, magenta) are co-localised in the cilia structure of the M5 nucleus of Schober (yellow arrows, merged image in panel c) by confocal imaging. Some centrifugal retinal neurons present a cilia structure *via* a ventricular dendrite (**d**, green). Some cilia of centrifugal retinal neurons show bPPL immunoreactivity (**e**, magenta). The merged image is presented in panel f. White arrows show the bPPL immunoreactive cilia structure. Scale bars = 10 μm (**c**) and 25 μm (**f**).

We also investigated immunohistochemically if in a second species, the brook lamprey (*L. reissneri*), centrifugal retinal neurons in M5NS also express bPPL and P-opsin (Fig. 6). In brook lamprey, some cilia structures in M5NS were stained by both anti-bPPL and anti-P-opsin antisera, although the levels of immunostaining varied among cilia (Fig. 6a–c, Supplementary Fig. S4). Retrograde tracing from the optic nerve of the brook lamprey also labelled the centrifugal retinal neurons in M5NS (Fig. 6d). Some centrifugal retinal neurons presented a cilia structure *via* a ventricular dendrite. Remarkably, some cilia of centrifugal retinal neurons were stained by anti-bPPL antiserum (Fig. 6d–f). These results with brook lamprey indicate that some centrifugal retinal neurons directly projecting to the retina express bPPL in their cilia. Our finding of a neural connection between M5NS cells expressing opsins and the retina could suggest the modulation of the retinal activity by brain photoreceptors.

## Discussion

In this study, we isolated and characterised a parapinopsin-like opsin, bPPL, from the Japanese river lamprey, *L. camtschaticum*. Phylogenetic analysis revealed that bPPL and one parapinopsin-related opsin from the Australian pouched lamprey, *Geotria australis*, (referred to as parapinopsin 53253-1-3^26^, accession no. ANV21069) form a clustered PPL (PP-like) group (Fig. 1). Moreover, the pouched lamprey has another parapinopsin-like gene (referred to as parapinopsin 53253-2-1^26^, accession no. ANV21070). We searched genomic DNA fragments of the orthologue of the pouched lamprey parapinopsin 53253-2-1 in the river lamprey genome database^35^. We then found the genomic DNA fragment encoding the full-length sequence of a parapinopsin-like opsin (LETJA | g14614, LETJA | g14615). Its predicted amino acid sequence exhibits about 88% identity to pouched lamprey parapinopsin 53253-2-1, suggesting that the river lamprey displays at least two types of parapinopsin-like opsins (PPL group), in addition to a ‘classical’ parapinopsin gene (PP group). Taken together, it is presumed that the diversification of opsins in the PPL group occurred before the diversification of lampreys, since both the river and pouched lampreys have two types of parapinopsin-like opsins.

The phylogenetic tree suggests that the divergence of PP and PPL groups occurred before the divergence of lamprey and gnathostome lineages, since ‘classical’ parapinopsin genes that are classified into the PP group are widely found in lamprey and gnathostomes. Interestingly, parapinopsin of elephant shark (*Callorhinchus milii*) is classified into the PPL group in the phylogenetic tree (Fig. 1). The members of the PP group are found in teleost, frogs and lizards, whereas the PPL group members are not found in their genome databases. Opsin genes of the PPL group might have been lost in the osteichthyan lineage. On the other hand, the classical parapinopsin genes are not found in the genome database of the elephant shark, suggesting that it was lost during the evolutionary process.

In general, opsins vary in spectral sensitivity and activating G protein subtypes. Our result suggests that bPPL activates Gi-type G protein, which decreases the cAMP level upon activation, in cultured cells (Fig. 2a), similarly to lamprey and teleost parapinopsins as reported previously^13^. On the other hand, we found that bPPL is a violet to blue-sensitive opsin (Fig. 2b,c) in contrast to the absorption characteristic of parapinopsin as a UV light-sensitive opsin^14^. The diversification of parapinopsins might be involved in the acquisition of different spectral sensitivities of the parapinopsin-related opsins, and then these parapinopsin-related opsins might be utilized for different physiological functions.

In two lamprey species, the river lamprey (*L. camtschaticum*) and brook lamprey (*L. reissneri*), bPPL was co-localised with P-opsin in M5NS cells, and some bPPL-expressing cells projected directly to the retina as centrifugal retinal neurons. However, the detailed morphological characteristics of bPPL-expressing cells were not identical between the species. In the river lamprey, bPPL is co-expressed with P-opsin in three kinds of M5NS cells localised in different areas (Areas I, II and III). Non-ciliary cells in Area III project axons to the retina, and the ciliary cells in Area I might form synapses with centrifugal retinal neurons (Fig. 5). On the other hand, some centrifugal retinal neurones in the brook lamprey exhibit morphological characteristics of cerebrospinal fluid (CSF)-contacting neurons and possess cilia. bPPL is co-localised with P-opsin in the cilia (Fig. 6), suggesting that light information captured by bPPL and P-opsin might directly transmit to the retina. In a previous study, some centrifugal retinal neurons of larval lamprey exhibited morphological characteristics of CSF-contacting neurons, with dendrites displaying club-like terminals protruding into the third ventricle^36^. This previous report also indicated that the proportion of CSF-contacting neurons decreases as the larvae grow. In this study, we used young adults of the brook lamprey and, therefore, characterised CSF-contacting neurons as centrifugal retinal neurons in the M5NS. These observations, together with a previous report that M5NS contains centrifugal retinal neurons projecting to the retina^33,34,36^, suggest that M5NS cells expressing both bPPL and P-opsin might serve as deep brain photoreceptors that directly and indirectly transmit the light information to the retina and might be involved in the control of the retinal photoreceptive function in the lampreys. To our knowledge, this finding is the first to suggest that deep brain photoreception is involved in the regulation of retinal photoreception, e.g. vision.

P-opsin, which is co-localised with bPPL in lampreys (Figs 4, 6), is classified into VA/VAL opsin group^28^. Several studies reported that VA/VAL opsins are blue to green sensitive and are localised in the brain of many teleost and birds^7–10,37^. We confirmed the absorption spectrum of P-opsin, with an absorption maximum at around 500 nm (Supplementary Fig. S5). This absorption spectrum is similar to teleost VA/VAL opsins^7,37^. VA/VAL opsins might serve as common blue to green-sensitive non-visual opsins in lower vertebrates. Co-localisation of bPPL and P-opsin in M5NS provides alternative possibilities for M5NS photoreception, either light sensitivity in a wide range of wavelength or generation of colour opponency, such as the pinopsin and parietopsin system in lizard parietal eyes^6^. Further studies are necessary to demonstrate the physiological role of this co-localisation.

Previous histological studies indicate that M5NS is innervated by optic fibres that terminate in the tegmental accessory optic area (TAOA) and project to the retina. This pattern establishes a feedback loop (retina-TAOA-M5NS-retina)^34^. Centrifugal retinal fibres project to and contact mainly the amacrine cells and some retinal ganglion cells^34,38^. Therefore, the light information generated in the retina may be modified in M5NS and be returned to the retina. Deep brain photoreceptor cells expressing bPPL and P-opsin might be involved in such feedback loops by adding environmental light information to the feedback signal. The adult lamprey exhibit negative phototaxis and dorsal light response as a vision-dependent behaviour^39^. Further, studies on such light-dependent behaviours involving ocular and extraocular photoreception could provide a clue to the physiological relevance. Such behavioural investigations, together with physiological studies on neural connections, might also provide insight into the ecological relevance of functional connections between the retina and opsin-expressing cells in the M5NS. The two lampreys used in this study spawn in rivers, although their lifestyles are different. River lampreys migrate and are parasitic on fishes as adults. Brook lampreys are non-migrating and non-parasitic. Thus, another issue to investigate is whether and how similarities and differences in the neural connections reflect similarities and differences in their lifestyles.

Centrifugal retinal neurons are found in various vertebrates, including teleost and birds, although their physiological characteristics are unclear^33,34,40^. Previous studies reveal that centrifugal retinal neurons are located in the mesencephalon in birds and may be related to visual information processing, for example, visual attention^41,42^. Our finding of neural connections between M5NS cells expressing the opsins and retina suggests the modulation of retinal activity by brain photoreceptors. Further studies are required to understand the detailed electrophysiological modification of retinal light information in M5NS.

## Methods

### Ethics statement

This experiment was approved by the Osaka City University animal experiment committee (#S0032) and complied with the Regulations on Animal Experiments from Osaka City University.

### Animals

Japanese river lampreys, *Lethenteron camtschaticum*, formerly known as *Lampetra japonica* or *Lethenteron japonicum*, were captured in the Ishikari River in Hokkaido, Japan, and were obtained commercially. Far Eastern brook lampreys, *Lethenteron reissneri*, were captured in the river near Lake Biwa, Japan.

### Isolation of cDNAs

Total RNA was extracted from the lamprey pineal organ, retina and brain using Sepasol(R)-RNA I (Nacalai Tesque) and reverse-transcribed to cDNAs using an oligo(dT) primer and SuperScript III (Invitrogen). cDNAs were used as templates for PCR amplification. A partial cDNA of bPPL was isolated from the lamprey brain by PCR amplification using the following degenerate primers: 5′-GCGGATCCICCIITNNTNGGNTGG-3′, corresponding to the amino acid sequence PP(F/L/V/I/M)(F/L/V/I/M)GW for the sense primer, and 5′-GCGAATTCIIIGC(A/G)TANGGN(C/A/G)NCCA-3′, corresponding to WXPYAX for the antisense primer. PCR amplification was performed at annealing temperatures of 40 °C or 46 °C. A partial cDNA of P-opsin was isolated from the lamprey brain *via* PCR amplification with gene-specific primers designed according to the genome sequences of the sea lamprey *Petromyzon marinus* P-opsin (accession no. U90671). The full-length cDNAs of these opsins were obtained using 3′ RACE and 5′ RACE systems (Invitrogen).

### Phylogenetic tree inference

Multiple alignment of the amino acid sequences of opsins, including lamprey bPPL, was performed with the XCED software^43^. Unambiguously aligned amino acid positions were subjected to phylogenetic analysis based on the neighbour-joining method with a simple Poisson correction^44^. Bootstrap analysis was conducted by the method of Felsenstein^45^.

The accession numbers of the sequence data from the DDBJ/EMBL/GenBank database are as follows: human Mel-1a melatonin-R (receptor), U14108; human M1 muscarinic acetylcholine-R (receptor), Y00508; human OPN4, AF147788; human OPN5, BC126198; human rgr, U15790; human peropsin, AF012270; human OPN3, AF140242; zebrafish parietopsin, XM_003201434; clawed frog parietopsin, NM_001045791; lizard parietopsin, DQ100320; iguana parietopsin, AB626970; elephant shark parapinopsin, AFP03346; pouched lamprey parapinopsin (referred to as 53253-1-3^26^), ANV21069; pouched lamprey parapinopsin (referred to as 53253-2-1^26^), ANV21070; river lamprey bPPL, AB116385; brook lamprey bPPL, LC500593; river lamprey parapinopsin, AB116380; clawed frog parapinopsin, AB159672; anole parapinopsin, AB626968; iguana parapinopsin, AB626969; spotted gar parapinopsin, ENSLOCP00000017452; catfish parapinopsin, AF028014; zebrafish PP1, AB626966; pufferfish PP1, AB626964; rainbow trout PP1, AB159673; zebrafish PP2, AB626967; pufferfish PP2, AB626965; rainbow trout PP2, AB675727; Ci-opsin1, AB058682; marine lamprey P-opsin, U90671; river lamprey P-opsin, LC500595; brook lamprey P-opsin, LC500594; clawed frog VA opsin, ACJ61344; chicken VAL opsin, ACX32474; chicken VA opsin, ABM66817; zebrafish valop-A, BAA94288; zebrafish valop-B, AAY56361; toad pinopsin, AF200433; chicken pinopsin, U15762; river lamprey red, AB116381; chicken iodopsin, X57490; goldfish red, L11867; mouse green, AF011389; human red, AH005298; human green, AH005296; goldfish UV, D85863; chicken violet, M92039; mouse UV, U92562; human blue, AH003620; goldfish blue, L11864; chicken blue, M92037; goldfish green, L11866; chicken green, M88178; river lamprey rhodopsin, AB116382; goldfish rhodopsin, L11863; chicken rhodopsin, D00702; mouse rhodopsin, M55171; human rhodopsin, U49742.

### GloSensor assay

The changes in the intracellular cAMP concentration of pigment-expressing HEK293S cells were measured using the GloSensor cAMP assay (Promega), as previously reported^13,29^. Briefly, cDNAs of lamprey bPPL were tagged with the epitope sequence for the monoclonal Rho1D4 antibody (ETSQVAPA). Tagged cDNA was inserted into the plasmid vector, pcDNA3.1 (Invitrogen). The expression constructs for bPPL were co-transfected with the pGloSensor-22F cAMP plasmid (Promega), and the transfected cells were incubated overnight in culture medium containing 10% fetal bovine serum (FBS) with 11-*cis*-retinal. Before the measurement, culture medium was replaced with a CO_2_-independent medium containing 10% FBS and 2% GloSensor cAMP Reagent (Promega). After equilibration with the medium and obtention of a steady basal signal, the cells were treated with 3.5 μM forskolin, a direct activator of adenylyl cyclase, to increase the intracellular cAMP levels. Luminescence, representing the amount of cAMP, was measured at 25 °C using a GloMax 20/20n Luminometer (Promega). To measure the light-induced change in cAMP levels, the transfected cells were irradiated with blue LED light for 5 sec. The relative response curve of lamprey bPPL was analysed using the GloSensor cAMP assay without forskolin following the previously described method^29^.

### *In situ* hybridisation

Brains were isolated from animals. The samples were fixed in 4% paraformaldehyde in 0.1 M sodium phosphate buffer (PB, pH 7.4) overnight at 4 °C. Each organ was cryoprotected by immersion in 0.1 M PB containing 15% and 30% sucrose, embedded in OCT compound (Sakura) and sectioned at 20 μm on a cryostat.

Digoxigenin-labelled antisense RNA probes for lamprey bPPL and P-opsin were synthesised using a DIG RNA Labelling Kit (Roche Applied Science), as previously reported^13,46^. In brief, sections were pre-treated with proteinase K and hybridised with the antisense RNA probe diluted in ULTRAhyb-Ultrasensitive Hybridisation Buffer (Ambion) at 68 °C overnight. The probe was detected using alkaline phosphatase-conjugated anti-digoxigenin (RRID: AB_2734716, Roche Applied Science), followed by a blue 5-bromo-4-chloro-3-indolyl phosphate nitro blue tetrazolium colour reaction.

### Antibodies

A rabbit polyclonal antiserum to the river lamprey bPPL and a mouse polyclonal antiserum to the river lamprey P-opsin were generated against N-terminal peptide sequences as follows: MSTPPLNVSDEERLSANGS for bPPL and MDALQESPPSHHSLPSALP for P-opsin. Specific immunoreactivity of each antiserum was examined using lamprey bPPL- or P-opsin-expressing HEK293S cells, as described in the Supplementary Information.

### Immunohistochemistry

Immunohistochemical analyses were conducted, as previously reported^32^. In brief, tissue sections were incubated with primary antisera (diluted 1:500) and then with one of the following antibodies: Alexa Fluor 488-conjugated anti-mouse (RRID: AB_2534069); anti-rabbit IgG (RRID: AB_2576217); Alexa Fluor 594-conjugated anti-mouse (RRID: AB_2534073); and anti-rabbit IgG (RRID: AB_2534079) (diluted 1:500; Invitrogen) for immunofluorescent detection. We examined the stained sections under a fluorescence microscope (Leica DM6000 B, Leica Microsystems) and a confocal laser scanning microscope (Leica TCS SP8, Leica Microsystems).

### Retrograde labelling

Retrograde labelling was performed as described previously with the following modifications^32^. After decapitation, intact brains were carefully removed and transferred into oxygenated lamprey Ringer’s solution (138.6 mM NaCl, 2.82 mM KCl, 0.24 mM NaHCO_3_ and 2.07 mM CaCl_2_). The neuronal tracer, neurobiotin (Vector Laboratories), was applied to the optic nerves of the lamprey brains. After 30 min, excess tracer was rinsed away with Ringer’s solution, and the brain was incubated overnight at 4 °C in oxygenated Ringer’s solution. After incubation, brains were fixed in 4% paraformaldehyde in 0.1 M PB overnight at 4 °C and sectioned with a cryostat. The sections were incubated with Alexa Fluor 488- or 594-conjugated streptavidin for 5 h at room temperature to visualise the neuronal tracer. Additionally, immunohistochemistry was performed as described above for the immunofluorescent detection of lamprey bPPL.

### Data deposition

The sequences reported in this paper have been deposited in the DDBJ database [accession nos. AB116385, LC500593-LC500595]

## Supplementary information

Supplementary information.
