# Supplementary material for: The non-visual opsins expressed in deep brain neurons projecting to the retina in lampreys
Source: Sci Rep. 2020 Jun 15;10:9669. doi: 10.1038/s41598-020-66679-2 (PMC7295746; doi:10.1038/s41598-020-66679-2)
Supplement: Supplementary file 1 — Supplementary information. [file 41598_2020_66679_MOESM1_ESM.pdf]

Supplementary Information for

The non-visual opsins expressed in deep brain neurons projecting to the retina in lampreys

Emi Kawano-Yamashita*[1,2], Mitsumasa Koyanagi[1], Seiji Wada[1], Tomoka Saito[1], Tomohiro Sugihara[1], Satoshi Tamotsu[2], Akihisa Terakita*[1]

1: Department of Biology and Geosciences, Graduate School of Science, Osaka City University, 3-3-138 Sugimoto, Sumiyoshi-ku, Osaka 558-8585, Japan.

2: Department of Chemistry, Biology, and Environmental Science, Faculty of Science, Nara Women's University, Kitauoyanishi-machi, Nara 630-8506, Japan.

Supplementary Text

Materials and methods

RNA sequencing.

The brain tissues of the lamprey were divided into five portions, containing mainly pineal organs, telencephalon, diencephalon, mesencephalon and rhombencephalon, respectively. The total RNAs were extracted from each portion using Sepasol(R)-RNA I (Nacalai Tesque). mRNAs were purified with an Oligotex-dT30 Super mRNA Purification Kit (TaKaRa). cDNA libraries were constructed using an Ultra-low Input RNA Kit (Chrontech), and each cDNA library was sequenced by using Illumina HiSeq 2500. Analyses of fragments per kilobase of exon per million (FPKM) values of two parapinopsin genes were conducted using MASER pipelines that can serially process Trinity, Bowtie, eXpress and DEGseq output (http://cell-innovation.nig.ac.jp/maser/index_en.html). Briefly, all sequenced raw reads were used for *de novo* assembly, and contigs containing each parapinopsin sequence and their FPKM values were obtained. The mRNA expression level of each portion is presented as a relative value obtained by dividing the FPKM value of the contig containing the sequence of each parapinopsin by the FPKM value of the contig containing the glyceraldehyde 3-phosphate dehydrogenase (GAPDH) gene.

Checking immunoreactivity of antisera against opsins using cultured cells expressing the opsins.

Immunoreactivity of antisera to river lamprey bPPL and P-opsin was investigated using HEK 293 cells expressing bPPL or P-opsin. Cells expressing bPPL or P-opsin were fixed in 100% methanol for 5 min, treated with phosphate buffered saline containing 2% bovine serum albumin and 0.1% Tween-20 for 30 min at room temperature and incubated with primary rabbit and mouse antisera against bPPL and P-opsin, respectively (diluted 1:500) and Rho1D4 (hybridoma culture fluid) overnight at 4°C. Subsequently, cells were incubated with Alexa Fluor 488-conjugated anti-mouse IgG (RRID: AB_2534069) or Alexa Fluor 594-conjugated anti-rabbit IgG (RRID: AB_2534079) (diluted 1:500; Invitrogen).

Expression and Spectrophotometry of P-opsin.

The expression of P-opsin in HEK293S cells and its purification was performed as described previously [1]. Briefly, cDNA of river lamprey P-opsin was tagged with the epitope sequence for the monoclonal antibody, Rho1D4 (ETSQVAPA). Tagged cDNA was inserted into the plasmid vector, pcDNA3.1 (Invitrogen). Expressed P-opsin was incubated overnight with excess 11-*cis*-retinal to reconstitute the pigment. P-opsin pigments were then extracted with a detergent, 1% dodecyl β-D-maltoside, in 50 mM HEPES buffer (pH 6.5) containing 140 mM NaCl (buffer A). The absorption spectra of pigments were recorded at 10°C using a Shimadzu UV-2450 spectrophotometer (Shimadzu, Japan).

References

1. Koyanagi M, Kawano E, Kinugawa Y, Oishi T, Shichida Y, Tamotsu S, Terakita A: **Bistable UV pigment in the lamprey pineal**. *Proc Natl Acad Sci USA* 2004, **101**(17):6687-6691.
2. Garcia-Fernandez JM, Jimenez AJ, Gonzalez B, Pombal MA, Foster RG: **An immunocytochemical study of encephalic photoreceptors in three species of lamprey**. *Cell Tissue Res* 1997, **288**(2):267-278.

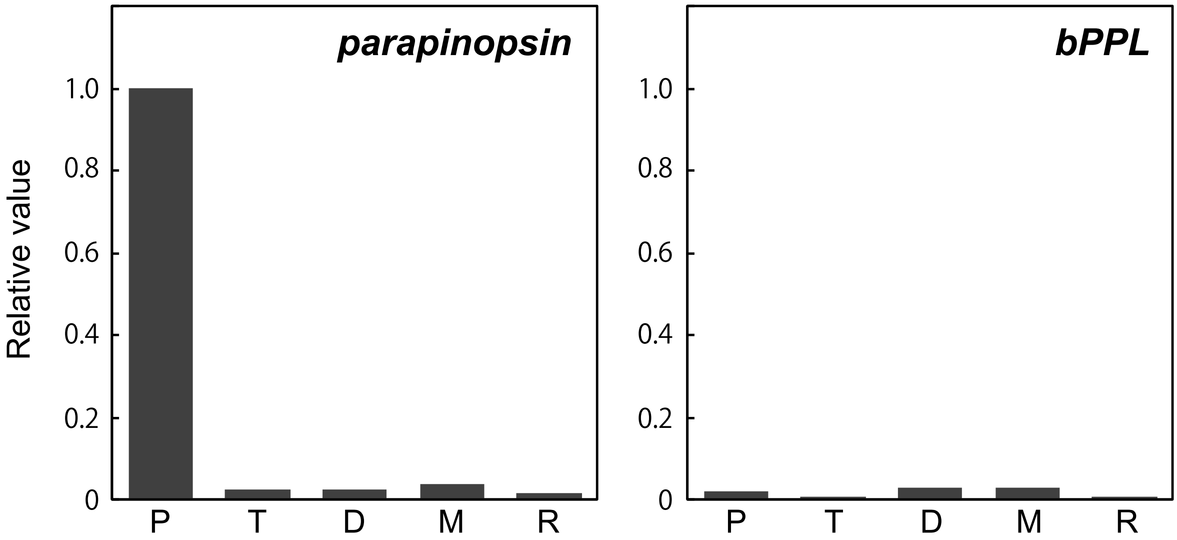

Supplementary Figure S1. Relative amounts of parapinopsin- and bPPL-mRNAs in the lamprey brain.

Parapinopsin is more abundantly distributed in the pineal organ-containing portion than other brain portions. bPPL is distributed in the diencephalon-, mesencephalon-, and pineal organ-containing portions and its mRNA level in each portion is much lower than parapinopsin in the pineal organ-containing portion. Relative values in each portion were calculated by dividing mRNA FPKM values of each opsin by GAPDH mRNA FPKM values as the internal standard. The level of parapinopsin in the pineal organ was referred to as 1.0. P: pineal organ, T: telencephalon, D: diencephalon, M: mesencephalon, R: rhombencephalon.

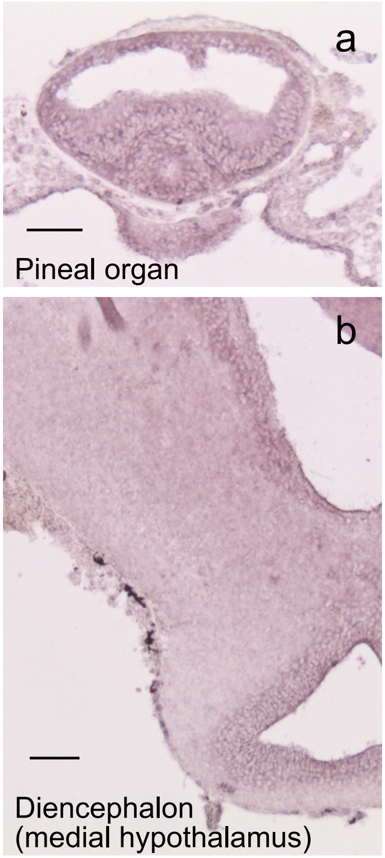

Supplementary Figure S2. *In situ* hybridisation of bPPL in the pineal organ and diencephalon of the river lamprey.

In situ hybridisation with the bPPL antisense probe shows that bPPL is not expressed in the pineal organ (a) or the medial hypothalamus of the diencephalon (b). Previous immunohistochemical study on anti-cone opsin antibody suggested that a deep brain photoreceptor exists in the medial hypothalamus, such as the nucleus of the postoptic commissure [2]. Therefore, opsin(s) other than bPPL might be expressed in the medial hypothalamus of the diencephalon. Scale bar = 100 μm.

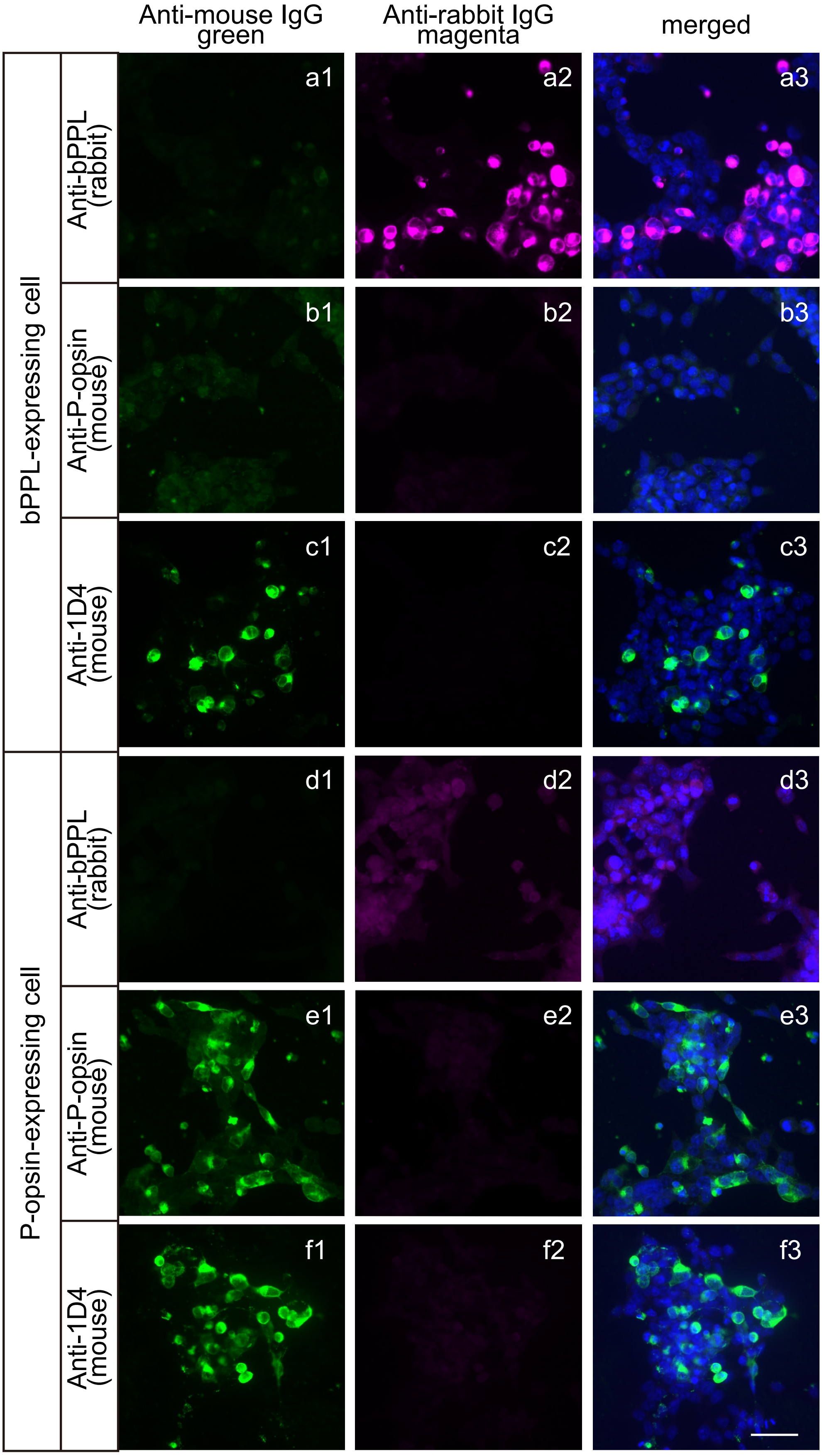

Supplementary Figure S3. The antibody specificities to bPPL and P-opsin.

HEK 293S cells expressing the river lamprey bPPL (a-c) and P-opsin (d-f) were treated with antisera against bPPL (a and d), P-opsin (b and e), and Rho1D4, a mouse monoclonal antibody against bovine rhodopsin C-terminal sequence (c and f). The bPPL or P-opsin C-terminus was tagged with Rho1D4 epitope sequence. Panels a1–f1 are anti-mouse IgG-treated images (green). Panels a2–f2 are anti-rabbit IgG-treated images (magenta). Panels a3–f3 are merged images in addition to the nuclear staining (blue). HEK293S cells expressing bPPL were immunostained by a rabbit polyclonal antiserum to bPPL and anti-rabbit IgG (a2). HEK293 cells expressing P-opsin were immunostained with anti-P-opsin antiserum and anti-mouse IgG (e1). Other combinations of first and secondary antibodies were immunonegative. Therefore, these antisera do not cross-react with other opsins. Scale bar = 50 μm.

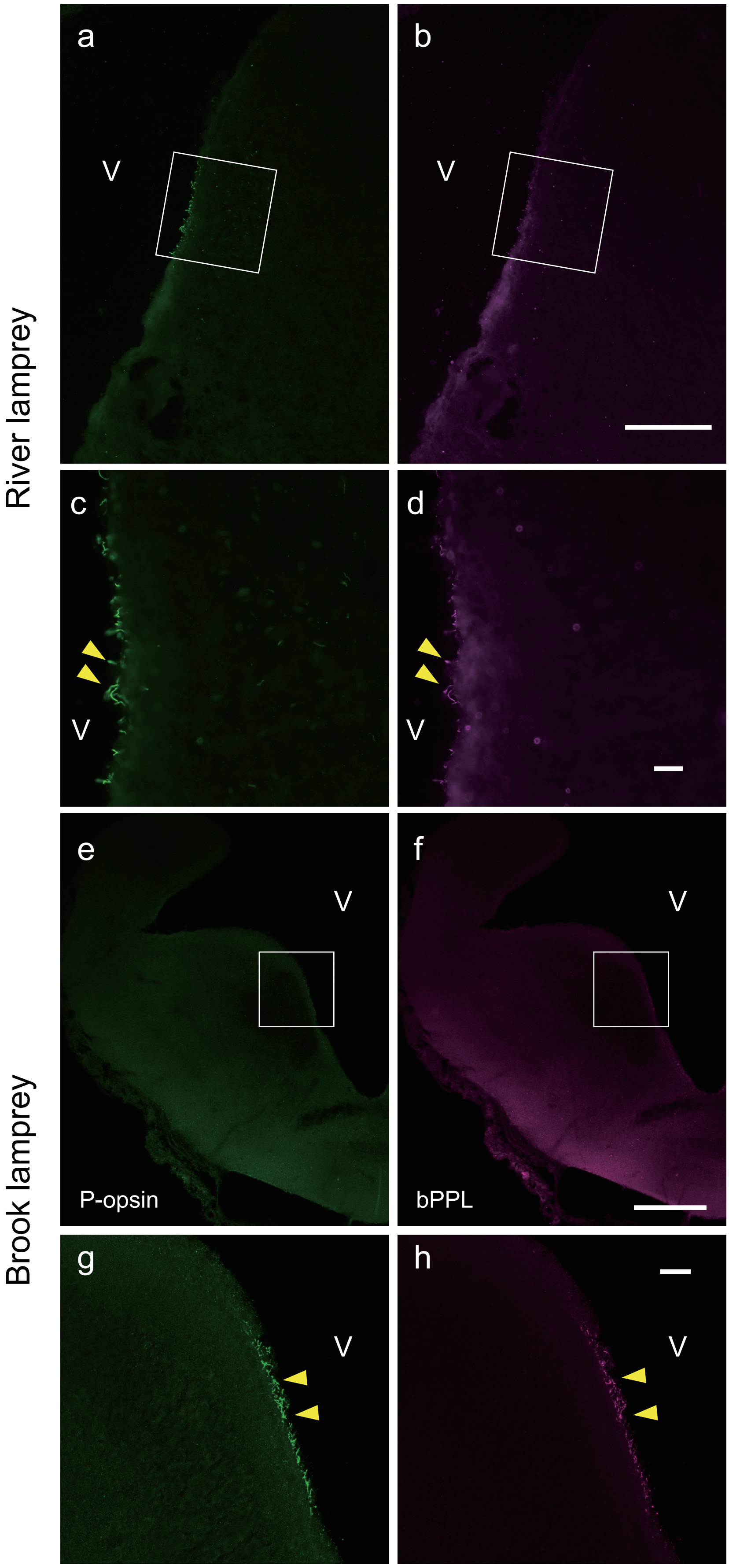

Supplementary Figure S4. Lower magnification images of the immunoreactivities against bPPL and P-opsin in M5NS of the river and brook lampreys.

Localisation of P-opsin (a, e; green) and bPPL (b, f; magenta) in the river lamprey (a, b) and the brook lamprey (e, f) is shown in low magnification views. P-opsin (c, g; green) and bPPL (d, h; magenta) are localised in the cilia structures of the M5NS (arrowheads) in river (c, d) and brook (g, h) lampreys. Panels c, d, g and h are higher magnification views of the areas indicated as boxes in panels a, b, e and f, respectively. V: third ventricle. Scale bars = 200 μm (b, f), 20 μ m (d, h).

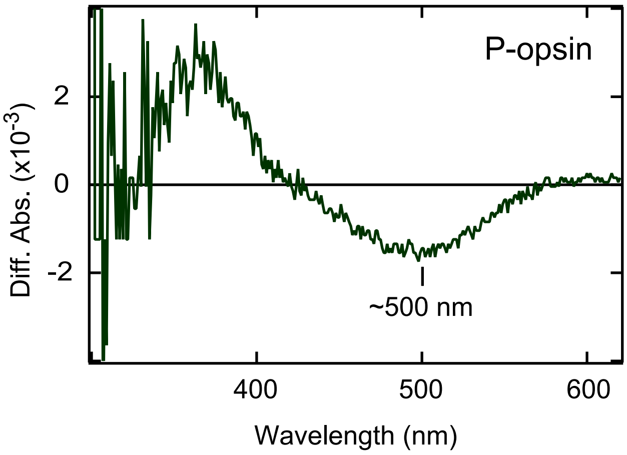

Supplementary Figure S5. The difference absorption spectrum of P-opsin.

The detergent extract of P-opsin-expressing cells was irradiated with green light in the presence of 5 mM hydroxylamine at pH 6.5. The difference spectrum of before minus after irradiation shows that the absorption maximum of P-opsin is at around 500 nm.
